# Hypocholesterolemia in liver and cardiovascular disease: Friend or foe?

**DOI:** 10.1111/joim.70142

**Published:** 2026-08-27

**Authors:** Valentina Flagiello, Emelie Barreby, Umberto Vespasiani‐Gentilucci, Stefano Romeo

**Affiliations:** ^1^ Operative Research Unit of Clinical Medicine and Hepatology Fondazione Policlinico Universitario Campus Bio‐Medico Roma Italy; ^2^ Centre for Reproduction, Metabolism and Molecular Medicine (CeRM), Department of Medicine (H7) Karolinska Institute Huddinge Sweden; ^3^ Research Unit of Hepatology, Department of Medicine and Surgery Università Campus Bio‐Medico di Roma Roma Italy; ^4^ Department of Molecular and Clinical Medicine University of Gothenburg Gothenburg Sweden; ^5^ Department of Experimental and Clinical Medicine University Magna Graecia of Catanzaro Catanzaro Italy; ^6^ Department of Endocrinology Karolinska University Hospital Huddinge Sweden; ^7^ Department of Cardiology Sahlgrenska University Hospital Gothenburg Sweden

**Keywords:** ApoB, cirrhosis, hypobetalipoproteinemia, hypocholesterolemia, steatosis

## Abstract

Hypocholesterolemia is perceived as benign given its association with lower cardiovascular risk. However, genetic and epidemiological evidence indicates that persistently low levels of low‐density lipoprotein cholesterol (LDL‐C) and apolipoprotein B (ApoB) arise from distinct biological mechanisms with different hepatic implications. Hypocholesterolemia may reflect either impaired hepatic assembly and secretion of ApoB‐containing lipoproteins or enhanced peripheral clearance of circulating lipoproteins, with divergent outcomes. Genetic defects disrupting ApoB‐dependent lipoprotein secretion, notably variants in apolipoprotein B gene (*APOB*) and microsomal triglyceride transfer protein gene (*MTTP*), limit hepatic lipid export and promote intrahepatic triglyceride retention, predisposing to steatosis and progressive liver disease without metabolic risk factors. Hypocholesterolemia driven by increased lipoprotein clearance, as observed with loss‐of‐function variants in proprotein convertase subtilisin/kexin type 9 (*PCSK9*) or angiopoietin‐like protein 3 (*ANGPTL3*), produces lifelong LDL‐C reduction that is cardioprotective without consistent evidence of adverse hepatic outcomes. Cohort and sequencing studies demonstrate an increased burden of liver disease in secretion‐driven hypocholesterolemia but not in clearance‐driven phenotypes. These findings indicate that clinical consequences are determined by the underlying biological mechanism rather than the degree of cholesterol lowering. After exclusion of secondary causes, genetic testing may be considered in people with metabolic dysfunction‐associated steatotic liver disease (MASLD) and hypocholesterolemia to clarify the mechanism, guide follow‐up, and enable family cascade screening. In impaired secretion phenotype forms, lifelong MASLD monitoring may be warranted, with timely consideration of therapy if liver disease progresses. In clearance‐mediated phenotypes, management should follow standard MASLD risk stratification and care, without additional surveillance related to the genetic defect.

## Graphical abstract

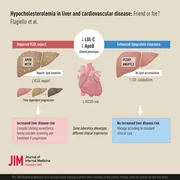

**Abbreviations**
ABL: abetalipoproteinemia
ANGPTL3: angiopoietin‐like protein 3
ANGPTL4: angiopoietin‐like protein 4
Apo A‐V: apolipoprotein A‐V
ApoB: apolipoprotein B
Apo C‐II: apolipoprotein C‐II
Apo C‐III: apolipoprotein C‐III
aOR: adjusted odds ratio
CAD: coronary artery disease
CHD: coronary heart disease
CI: confidence interval
EL: endothelial lipase
ER: endoplasmic reticulum
FHBL: familial hypobetalipoproteinemia
GLP‐1: glucagon‐like peptide‐1
HCC: hepatocellular carcinoma
HDL‐C: high‐density lipoprotein cholesterol
HR: hazard ratio
IDR: incidence density ratio
LDL‐C: low‐density lipoprotein cholesterol
LDLR: low‐density lipoprotein receptor
LDLRAP1: LDL receptor adaptor protein 1
LoF: loss‐of‐function
LPL: lipoprotein lipase
MASLD: metabolic dysfunction‐associated steatotic liver disease
MASH: metabolic dysfunction‐associated steatohepatitis
MTP: microsomal triglyceride transfer protein
MTTP: microsomal triglyceride transfer protein gene
MRI‐PDFF: magnetic resonance imaging‐proton density fat fraction
NASH: nonalcoholic steatohepatitis
NHLBI: National Heart, Lung, and Blood Institute
NITs: noninvasive tests
OR: odds ratio
PCSK9: proprotein convertase subtilisin/kexin type 9
PNPLA3: patatin‐like phospholipase domain‐containing 3
RR: relative risk
TG: triglyceride
THR‐β: thyroid hormone receptor‐β
TM6SF2: transmembrane 6 superfamily member 2
UKBB: UK Biobank
US: ultrasound
VLDL: very low‐density lipoprotein

## Introduction

In clinical practice, hypocholesterolemia is commonly perceived as a benign or even desirable biochemical state, largely because of its association with lower cardiovascular risk [1, 2, 3, 4]. However, previous work has questioned whether very low low‐density lipoprotein cholesterol (LDL‐C) concentrations should be interpreted uniformly as benign, emphasizing that the clinical meaning of low cholesterol depends on the biological context in which it occurs [5]. Low circulating cholesterol levels may arise from fundamentally distinct biological mechanisms, with markedly different consequences for organ‐specific disease, particularly for the liver [6, 7]. Indeed, hypocholesterolemia may reflect predominantly either impaired hepatic lipoprotein secretion or enhanced peripheral clearance, two mechanisms that differentially shape systemic lipid exposure and hepatic lipid handling, with distinct implications for organ‐specific risk [8, 9]. Notably, hypocholesterolemia is also a well‐recognized feature in advanced liver disease, where reduced hepatic synthesis, assembly, and secretion of apolipoprotein (Apo)B‐containing lipoproteins lead to low circulating cholesterol levels. In this context, low cholesterol reflects impaired hepatocellular function and is associated with worse clinical outcomes, representing a negative prognostic marker [10].

Hypocholesterolemia is clinically defined as persistently low circulating concentrations of ApoB‐containing lipoproteins, typically reflected by LDL‐C and/or ApoB levels below the 5th percentile for age and sex (with mean LDL‐C levels around 1.0 mmol/L [1.05 ± 0.43 mmol/L]), after exclusion of lipid‐lowering therapy and secondary causes, including hyperthyroidism, severe malnutrition, critical illness, active malignancy, and chronic liver disease [6, 7, 8, 10, 11]. Depending on the underlying phenotype and biological mechanism, hypocholesterolemia may also be associated with reduced circulating triglyceride (TG) levels and, in selected contexts, with concomitant changes in high‐density lipoprotein cholesterol (HDL‐C) [6].

Inherited forms are exemplified by abetalipoproteinemia (ABL) and familial hypobetalipoproteinemia (FHBL), which encompass a spectrum of phenotypes ranging from near‐complete absence of ApoB‐containing lipoproteins in biallelic disease to partial quantitative reductions in heterozygosity [6, 8]. Despite this heterogeneity, current clinical guidelines remain largely focused on the management of hypercholesterolemia [1], offering limited recommendations for the diagnostic work‐up and longitudinal assessment of people with primary hypocholesterolemia [2, 6]. However, although the genetic and biological bases of primary hypocholesterolemia are increasingly recognized, their translation into clinical management remains poorly defined. In particular, there is still no standardized framework to identify which individuals with persistently low LDL‐C and/or ApoB should undergo genetic testing, how liver involvement should be assessed at baseline, how frequently hepatic risk should be monitored over time, and when cascade evaluation of relatives should be considered. The therapeutic implications are even less established. Current management is largely extrapolated from experience in rare inherited lipid disorders, metabolic dysfunction‐associated steatotic liver disease (MASLD), or pharmacological studies of lipid‐lowering pathways, rather than from dedicated trials in people with impaired secretion phenotype hypocholesterolemia. As a result, it remains unclear whether interventions aimed at reducing intrahepatic lipid accumulation, improving hepatic lipid disposal, or modifying metabolic cofactors can alter liver‐related outcomes in this specific population. Prospective longitudinal studies and targeted interventional trials are therefore needed to define clinically meaningful endpoints, establish monitoring strategies, and determine whether mechanism‐based therapies can modify the natural history of liver disease associated with primary hypocholesterolemia.

## Physiology

### Hepatic lipoprotein secretion

One major biological mechanism underlying hypocholesterolemia is impaired hepatic lipoprotein secretion, a process that limits the export of TG‐rich lipoproteins from the hepatocyte and predisposes to intrahepatic lipid retention. At the cellular level, this mechanism reflects alterations in the intracellular pathways governing the assembly and secretion of very low‐density lipoproteins (VLDL) (Fig. 1).

**Fig. 1:**
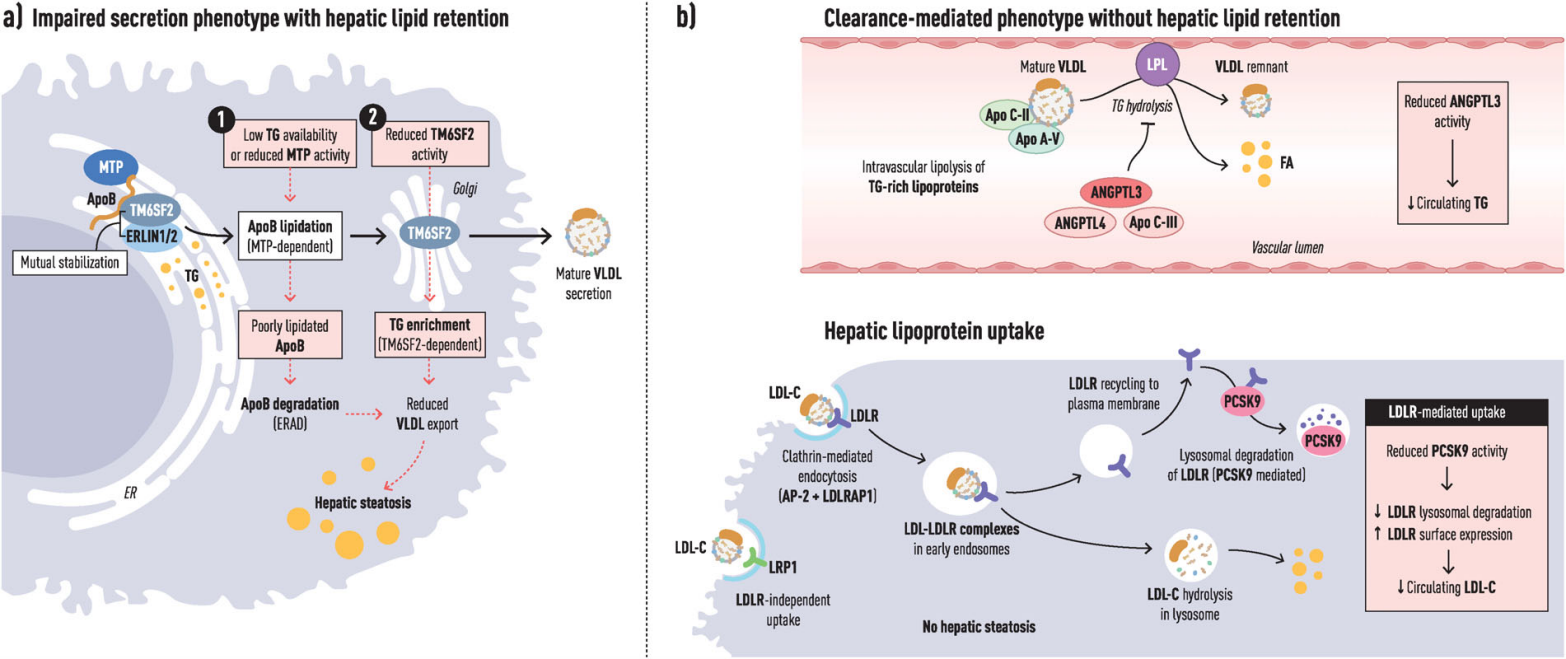
Pathophysiological mechanisms of hypocholesterolemia and divergent hepatic outcomes. (a) Impaired secretion phenotype with hepatic lipid retention. Reduced MTP activity, altered ER quality‐control pathways, or reduced TM6SF2 activity impair ApoB lipidation, TG enrichment, and mature VLDL export, leading to ApoB degradation and hepatic steatosis. Within the ER, TM6SF2 and the ERLIN1/2 complex reciprocally stabilize each other and contribute to ApoB stabilization, so that reduced TM6SF2 activity favors ApoB degradation. (b) Clearance‐mediated phenotype without hepatic lipid retention. Increased intravascular lipolysis of TG‐rich lipoproteins, as observed with reduced ANGPTL3 activity, lowers circulating TG. Increased hepatic LDL‐C uptake, as observed with reduced PCSK9 activity, decreases LDLR lysosomal degradation and increases LDLR surface expression, lowering circulating LDL‐C. ANGPTL3, angiopoietin‐like protein 3; ANGPTL4, angiopoietin‐like protein 4; AP‐2, adaptor protein complex 2; Apo A‐V, apolipoprotein A‐V; ApoB, apolipoprotein B; Apo C‐II, apolipoprotein C‐II; Apo C‐III, apolipoprotein C‐III; ER, endoplasmic reticulum; ERAD, ER‐associated degradation; ERLIN1/2, ER lipid raft protein 1 and 2; LDL, low‐density lipoprotein; LDL‐C, low‐density lipoprotein cholesterol; LDLR, low‐density lipoprotein receptor; LDLRAP1, LDL receptor adaptor protein 1; LPL, lipoprotein lipase; LRP1, LDL receptor‐related protein 1; MTP, microsomal triglyceride transfer protein; PCSK9, proprotein convertase subtilisin/kexin type 9; TG, triglyceride; TM6SF2, transmembrane 6 superfamily member 2; VLDL, very low‐density lipoprotein.

Assembly of VLDL is initiated in the endoplasmic reticulum (ER) through the synthesis of ApoB by microsomal TG transfer protein (MTP), encoded by the microsomal TG transfer protein gene (*MTTP*) [12]. MTP mediates early lipidation of nascent ApoB and prevents its proteasomal degradation [6]. When TG availability or MTP activity is insufficient, ApoB is targeted for ER‐associated degradation, thereby limiting VLDL secretion [13, 14]. Beyond initial lipidation, intracellular handling of ApoB within the ER is governed by quality‐control mechanisms that determine the balance between productive lipoprotein assembly and degradation [13]. The ER lipid raft‐associated protein 1/2 (ERLIN1/2) complex has been implicated in these pathways by modulating ER‐associated degradation, thereby influencing ApoB availability for VLDL assembly under conditions of limited lipidation capacity [15, 16]. ERLIN1/2 and transmembrane 6 superfamily member 2 (TM6SF2) reciprocally bind and stabilize one another, and within this complex, TM6SF2 directly binds and stabilizes ApoB through its two intraluminal loops, whereas ERLIN1/2 stabilize ApoB indirectly, by maintaining TM6SF2 levels [17]. Subsequent VLDL maturation involves further TG enrichment, a process in which TM6SF2 facilitates TG incorporation and efficient VLDL export [18, 19]. A second lipidation step occurs in the Golgi apparatus, resulting in fully mature VLDL particles competent for secretion into the circulation [13].

### VLDL cargo discharge and conversion to LDL‐C

In contrast to impaired hepatic secretion, hypocholesterolemia may also result from enhanced intravascular lipolysis, peripheral processing of TG‐rich lipoproteins, and increased LDL‐C clearance. These mechanisms reduce circulating lipid levels without promoting hepatic lipid retention.

After secretion into the circulation, VLDL particles undergo progressive TG hydrolysis, a process primarily mediated by lipoprotein lipase (LPL) at the capillary endothelium of adipose tissue and muscle, leading to the generation of remnant particles and ultimately LDL‐C [20, 21]. This pathway involves ApoB100‐containing VLDL as well as ApoB48‐containing intestinal lipoproteins, both contributing to remnant lipoprotein metabolism [22, 23]. LPL activity is tightly regulated by a set of apolipoproteins and accessory proteins that modulate the efficiency and tissue specificity of intravascular lipolysis [24, 25]. Apolipoprotein C‐II acts as an essential cofactor for LPL activation, whereas apolipoprotein A‐V facilitates the interaction between TG‐rich lipoproteins and the lipolytic process [26, 27, 28]. Effective localization of LPL to the endothelial surface requires glycosylphosphatidylinositol‐anchored high‐density lipoprotein‐binding protein 1 and lipase maturation factor 1, which are critical for LPL stability and function [29, 30]. In contrast, angiopoietin‐like proteins 3 and 4 (ANGPTL3 and ANGPTL4) and apolipoprotein C‐III inhibit LPL activity, and, in the case of ANGPTL3, also suppress endothelial lipase (EL), thereby modulating intravascular lipolysis, TG clearance, and the conversion of VLDL to LDL‐C [25, 31, 32, 33].

### Catabolism of LDL‐C

Beyond intravascular lipolysis, circulating LDL‐C is removed predominantly through receptor‐mediated hepatic uptake, a process that lowers plasma cholesterol levels without inducing hepatic lipid accumulation. Clearance of circulating LDL‐C is primarily mediated by receptor‐dependent hepatic endocytosis through the LDL‐C receptor (LDLR). Binding of apolipoprotein B‐100 on the LDL‐C surface to LDLR at the plasma membrane triggers clathrin‐mediated endocytosis via recruitment of the adaptor protein complex AP‐2 and LDL receptor adaptor protein 1 [34]. Internalized LDL–LDLR complexes are trafficked to early endosomes, where acidic pH promotes dissociation of LDL‐C from the receptor. LDLR is subsequently recycled to the cell surface, whereas LDL‐C particles are delivered to lysosomes for hydrolysis and cholesterol release [35]. The efficiency of this pathway is tightly regulated at multiple levels. LDLR abundance at the cell surface is controlled by transcriptional regulation and posttranslational mechanisms, most notably by proprotein convertase subtilisin/kexin type 9 (PCSK9), which binds LDLR and redirects it toward lysosomal degradation rather than recycling, thereby reducing LDL uptake [36]. Additional modulators of clathrin‐mediated endocytosis and endosomal trafficking contribute to fine‐tuning LDL‐C internalization and receptor recycling [35]. Emerging evidence implicates additional regulators of endocytic and vesicular trafficking in LDLR function, further refining the molecular framework governing hepatic LDL‐C clearance [37].

## Variants disrupting VLDL secretion: protection against cardiovascular risk but increase in chronic liver disease risk

Rare genetic defects impairing hepatic VLDL assembly and secretion define a distinct group of monogenic hypocholesterolemic disorders characterized by intrahepatic retention of ApoB‐containing lipoproteins, primarily caused by pathogenic variants in *APOB* and *MTTP* (Table 1) [6, 8]. Biallelic loss‐of‐function (LoF) variants in *MTTP* or *APOB*, classically described in ABL, correspond to the most severe phenotypes and are associated with a near‐complete absence of circulating ApoB‐containing lipoproteins [6, 38]. These biallelic conditions are exceptionally rare, with the incidence reported as less than 1 in 1,000,000 individuals [6, 39]. Homozygotes for LoF variants in these genes typically exhibit markedly reduced LDL‐C concentrations (often <0.1 mmol/L), total cholesterol around 0.9 mmol/L, and TG levels generally below 0.25 mmol/L, whereas HDL‐C remains relatively preserved or only modestly decreased [3]. Heterozygous pathogenic variants in *APOB*, leading to haploinsufficiency and defining FHBL, are substantially more prevalent in the general population, with an estimated prevalence ranging from 1 in 700 to 3,000 individuals based on observed and estimated reports [6]. These individuals display a milder phenotype, with mean LDL‐C levels around 1.0 mmol/L (1.05 ± 0.43 mmol/L) and TG levels approximately 0.48 mmol/L [0.34–0.70], whereas HDL‐C is largely preserved [6, 8].

**Table 1:** Effects of genetic variants affecting lipoprotein metabolism on circulating lipoproteins and organ involvement.

Pathophysiological mechanism	Gene (function)	Genetic condition	Effect on circulating lipoproteins	Effect on target organs (liver/heart)	Key evidence
Impaired hepatic lipoprotein secretion	***APOB*** Structural apolipoprotein required for assembly and secretion of VLDL (ApoB100) and chylomicrons (ApoB48)	Biallelic (homozygous or compound heterozygous) LoF ABL	LDL‐C ↓↓, ApoB absent, TG ↓↓, HDL‐C ↔/↓	**Liver**: increased risk of steatosis, fibrosis, cirrhosis and HCC **Heart**: reduced ASCVD risk	Case reports and family‐based series [8, 38, 48, 49, 50]
		Monoallelic (heterozygous) LoF FHBL	LDL‐C ↓, ApoB ↓, TG ↔/↓, HDL‐C ↔		Large population‐based cohorts and sequencing studies [9, 46, 47, 48, 51]
	***MTTP*** Endoplasmic reticulum lipid transfer protein essential for early lipidation of nascent ApoB and VLDL assembly	Biallelic (homozygous or compound heterozygous) LoF ABL	LDL‐C ↓↓, ApoB absent, TG ↓↓, HDL‐C ↔	**Liver**: increased risk of steatosis, fibrosis, cirrhosis and HCC **Heart**: reduced ASCVD risk	Case reports and multicenter clinical series [8, 38, 49, 52]
		Monoallelic LoF Heterozygous hypobetalipoproteinemia spectrum	LDL‐C ↔/↓, ApoB ↔, TG ↔, HDL‐C ↔		Very limited human data [53, 54, 55, 56]
Enhanced lipoprotein clearance	***PCSK9*** Regulator of LDL‐C receptor degradation controlling hepatic clearance of LDL‐C particles	Biallelic (homozygous or compound heterozygous) LoF PCSK9 deficiency	LDL‐C ↓↓, ApoB ↓↓, TG ↔, HDL‐C ↔	**Liver**: no evidence of increased hepatic risk **Heart**: reduced ASCVD risk	Population‐based and family studies [47, 58, 63, 64]
		Monoallelic LoF Heterozygous PCSK9 loss‐of‐function	LDL‐C ↓, ApoB ↓, TG ↔, HDL‐C ↔/↓		Large population‐based cohorts, Mendelian randomization studies, randomized controlled trials [58, 64, 65, 66]
	***ANGPTL3*** Secreted inhibitor of LPL and EL regulating intravascular lipoprotein catabolism	Biallelic (homozygous or compound heterozygous) LoF Familial combined hypolipidemia	LDL‐C ↓↓, ApoB ↓↓, TG ↓↓, HDL‐C ↓	**Liver**: no consistent evidence of increased hepatic risk **Heart**: reduced ASCVD risk	Family‐based studies and targeted clinical investigations [59, 60, 62, 69]
		Monoallelic LoF Heterozygous ANGPTL3 loss‐of‐function	LDL‐C ↓, TG ↓, HDL‐C ↔/↓		Population‐based cohorts and genetic association studies [31, 32, 62]

*Note*: The main genetic causes of primary hypocholesterolemia are summarized according to the underlying pathophysiological mechanism, distinguishing disorders characterized by impaired hepatic lipoprotein secretion from those driven by enhanced lipoprotein clearance. For each gene, the associated genetic condition, the effects on circulating lipoproteins, and the predominant involvement of target organs (liver and heart) are reported, together with the level of available evidence.

Abbreviations: ABL, abetalipoproteinemia; *ANGPTL3*, angiopoietin‐like protein 3; ApoB, apolipoprotein B; *APOB*, apolipoprotein B gene; ASCVD, atherosclerotic cardiovascular disease; EL, endothelial lipase; FHBL, familial hypobetalipoproteinemia; HCC, hepatocellular carcinoma; HDL‐C, high‐density lipoprotein cholesterol; LDL‐C, low‐density lipoprotein cholesterol; LoF, loss‐of‐function; LPL, lipoprotein lipase; *MTTP*, microsomal triglyceride transfer protein; *PCSK9*, proprotein convertase subtilisin/kexin type 9; TG, triglyceride; VLDL, very low‐density lipoprotein.

Common genetic variants in the general population, such as patatin‐like phospholipase domain‐containing 3 (*PNPLA3*) and *TM6SF2*, are well‐established determinants of hepatic fat accumulation and disease progression [40]. In the context of impaired VLDL secretion due to *APOB* or *MTTP* LoF, these variants may further promote intrahepatic lipid retention and liver injury. In a familial setting, the coexistence of a heterozygous *APOB* truncating variant with homozygous *PNPLA3* p.I148M and heterozygous *TM6SF2* p.E167K has been reported to co‐segregate with a steatotic liver disease phenotype progressing to cirrhosis and hepatocellular carcinoma (HCC), suggesting a potential contribution of combined genetic defects in disease expression within affected pedigrees [41, 42, 43]. However, current evidence does not support a clear modifying effect at a population level [9]. In people with MASLD homozygous for *PNPLA3* p.I148M, disease severity does not differ according to *APOB* variant carriage [9]. Consistently, the hepatic harmful effect of *APOB* variants appears to be more evident in individuals with a low polygenic score for hepatic fat content, presumably because those with a higher score already have an intrinsically elevated hepatic risk [9].

### Effect of loss of function variants in *APOB* and cardiovascular disease

Family and population‐based genetic studies investigating carriers of truncating variants in *APOB* indicate a protective cardiovascular phenotype associated with lifelong exposure to very low concentrations of ApoB‐containing lipoproteins [44]. In a heterozygous carrier of an *APOB* truncating variant (c.6943G>T) generating a shortened ApoB‐51 isoform, markedly reduced lipid levels (LDL‐C 44 mg/dL; ApoB 0.3 g/L) were associated with absence of coronary atherosclerosis at angiography and freedom from major cardiovascular events during a 20‐year follow‐up, despite the presence of multiple traditional cardiovascular risk factors [45]. Family‐based case–control observations provided early supportive signals, showing lower carotid arterial stiffness—a surrogate vascular marker—in people with FHBL after exclusion of those with diabetes, despite broadly comparable traditional cardiovascular risk factors between groups [46].

In a meta‐analysis integrating family‐based sequencing and 12 case–control studies, including 57,973 people (18,442 with early‐onset coronary heart disease [CHD] and 39,531 controls), carriers of rare *APOB* truncating variants had 43 mg/dL lower LDL‐C, 30% lower TG levels, and a 72% lower risk of CHD compared with noncarriers (odds ratio [OR] 0.28; 95% confidence interval [CI] 0.12–0.64; *p* = 0.002) [44]. Large prospective sequencing analyses integrating population‐based cohorts, including the UK Biobank (UKBB) and five longitudinal studies from the National Heart, Lung, and Blood Institute (NHLBI), further strengthened this association [47]. Protein‐truncating variants in *APOB* (and *PCSK9*) were associated with 49 mg/dL lower estimated untreated LDL‐C levels in the NHLBI cohorts, and 45 mg/dL lower levels in the UKBB compared with noncarriers, together with reduced lifetime exposure to LDL‐C [47]. Across 209,537 people followed prospectively, variant carriers showed a 49% lower risk of incident CHD compared with noncarriers (HR 0.51; 95% CI 0.32–0.81; *p* = 0.004) [47]. Taken together, these findings demonstrate that hypocholesterolemia driven by *APOB* truncating variants is associated with cardiovascular protection, supporting the concept that lifelong reduction of ApoB‐containing lipoproteins is linked to lower coronary risk.

### Effect of loss of function variants in *APOB* and liver disease

In contrast to the cardiovascular protection consistently observed in carriers of *APOB* truncating variants, early evidence linking primary hypocholesterolemia to liver disease emerged from small clinical cohorts and family‐based studies of FHBL and was subsequently supported by population‐based genetic studies [7, 9, 48, 49, 50, 51]. In adults with FHBL caused by *APOB* truncating variants, hepatic steatosis was frequently reported, with documented cases of liver fibrosis and occasional cirrhosis, suggesting that impaired hepatic VLDL export promotes intrahepatic lipid retention [48]. In a retrospective comparative analysis integrating clinical, imaging and histological data from published ABL probands, hepatic steatosis and fibrosis was observed in both conditions [49]. Liver abnormalities were described as independent of total cholesterol, TG and ApoB levels, and in ABL also independent of ApoB truncation size [49]. Heterozygous pathogenic variants in *APOB*, defining FHBL, are substantially more prevalent than biallelic forms and represent a clinically relevant model of chronic partial impairment of ApoB secretion [8]. These people typically display persistently reduced LDL‐C and ApoB concentrations while remaining otherwise asymptomatic [8]. Family‐based investigations in FHBL kindreds have reported co‐segregation of *APOB* truncating variants with hepatic steatosis and, in selected families, with cirrhosis and HCC [50]. In a large kindred carrying the p.K2240X variant, low cholesterol and hepatic steatosis were observed in 10 family members, 1 individual had cirrhosis, and 4 died of HCC or carcinoma arising on cirrhosis, consistent with autosomal dominant co‐segregation of low cholesterol and liver disease [50].

At the population level, in a study conducted in the UKBB and CONSTANCES cohorts (94,966 and 34,653 participants, respectively), people with LDL‐C levels below the 5th percentile for age and sex were identified and subsequently genetically characterized [7]. Carriers of rare *APOB* LoF variants (7680 and 5655 person‐years of follow‐up, respectively) showed an increased risk of cirrhosis complications (UKBB IDR 6.20, 95% CI 2.31–16.6, *p* = 0.0045; CONSTANCES IDR 6.05, 95% CI 3.24–11.3, *p* < 0.0001) and overall hepatic complications (UKBB IDR 4.19, 95% CI 1.74–10.1, *p* = 0.0077; CONSTANCES IDR 4.31, 95% CI 2.38–7.80, *p* < 0.0001) [7]. The association remained significant after adjustment for conventional metabolic risk factors, supporting impaired hepatic lipoprotein secretion rather than low LDL‐C levels per se as the underlying mechanism [7].

A comprehensive study integrating clinical cohorts of participants with cirrhosis and HCC, formal family segregation analyses, and large population‐based sequencing data further consolidated the causal link between *APOB* LoF variants and progressive liver disease by quantifying different stages of hepatic involvement, from steatosis and fibrosis in family‐based analyses to cirrhosis and HCC in population‐based cohorts [9]. In a case–control study of severe MASLD (defined as advanced fibrosis or HCC; cases = 510 and controls = 261), rare *APOB* variants were enriched more than six‐fold in cases compared to controls (4.5% vs. 0.7%) and conferred an almost 14‐fold higher risk after adjustment for age and sex for severe MASLD (adjusted odds ratio [aOR] 13.8, 95% CI 2.7–70.7; *p* = 0.002) [9]. Within pedigrees of probands with severe MASLD, family‐based analyses in 11 families from Milan, Italy including 32 first‐degree relatives showed hepatic steatosis in 12/32 (37.5%) relatives and liver fibrosis in 7/32 (21.9%) relatives, with co‐segregation of *APOB* variants with liver steatosis (transmitted [T] vs. untransmitted [U] alleles; T:U 7:2; *p* = 0.004), severe steatosis (T:U 5:0; *p* = 0.003), and liver fibrosis (T:U 4:1; *p* = 0.034) [9].

When extended through a meta‐analysis, including 18 pedigrees and 100 relatives, over‐transmission of *APOB* LoF variants in people with liver fibrosis remained significant (T:U 5:1; *p* = 0.020, transmission disequilibrium test) [9]. Replication at the population level in the million veteran program and UKBB confirmed that *APOB* LoF carriers have an increased risk of cirrhosis (OR 3.28; *p* = 0.009) and even higher risk of HCC (OR 11.68; *p* = 6.35 × 10^−5^), with consistent effects across European and African Americans [9].

Despite the increased risk observed at the population level, the observed penetrance of severe liver outcomes among *APOB* LoF carriers appears incomplete and variable, likely influenced by additional genetic and metabolic modifiers. We therefore examined the penetrance of cirrhosis and HCC in carriers of ApoB rare LoF in 417,657 participants from the UKBB. *APOB* LoF variants were observed in 1,113 participants (0.27%, corresponding to 1 in 375). Cirrhosis was present in 19/1,113 *APOB* LoF carriers (observed carrier risk 1.71%) compared with 3,336/416,544 non‐carriers (0.80%), corresponding to an absolute risk difference of +0.91 percentage points and a relative risk (RR) of 2.13. HCC was present in 5/1113 *APOB* LoF carriers (0.45%) compared with 472/416,544 noncarriers (0.11%), corresponding to an absolute risk difference of +0.34 percentage points and an RR of 3.96. These estimates should be interpreted as observed UKBB cohort risks rather than lifetime penetrance, and the HCC estimate requires caution because it is based on only five affected *APOB* LoF carriers (Table 2). With a frequency of 1 in 375, the frequency in homozygosity would be approximately 1 in 563,000.

**Table 2:** Prevalence of apolipoprotein B gene (*APOB*) loss‐of‐function (LoF) variants and observed liver outcome risk in UK Biobank (UKBB).

Outcome	Cases in cohort, *n*/*N* (%)	*APOB* LoF carriers in cohort, *n*/*N* (%)	*APOB* LoF carriers among cases, *n*/*N* (%)	*APOB* LoF carriers among controls, *n*/*N* (%)	Observed carrier risk, *n*/*N* (%)	Noncarrier risk, *n*/*N* (%)	Absolute risk difference, percentage points	Relative risk carriers vs. noncarriers
Cirrhosis	3,355/417,657 (0.80)	1,113/417,657 (0.27)	19/3,355 (0.57)	1,094/414,302 (0.26)	19/1,113 (1.71)	3,336/416,544 (0.80)	+0.91	2.13
HCC	477/417,657 (0.11)	1,113/417,657 (0.27)	5/477 (1.05)	1,108/417,180 (0.27)	5/1,113 (0.45)	472/416,544 (0.11)	+0.34	3.96

*Note*: Data are derived from a UKBB analysis of *APOB* LoF carriers. Observed carrier risk represents disease risk among *APOB* LoF carriers in the analyzed cohort and should not be interpreted as lifetime penetrance. Relative risks are crude ratios derived from carrier and noncarrier counts. The HCC estimate should be interpreted cautiously because it is based on only five affected *APOB* LoF carriers.

Abbreviations: *APOB*, apolipoprotein B gene; HCC, hepatocellular carcinoma.

Despite this increased hepatic risk, carriers displayed reduced LDL‐C and TG levels and were protected against coronary artery disease (CAD), highlighting a marked phenotypic dissociation between cardiovascular and hepatic outcomes [9].

Interestingly, stratification by the variant position further refined this phenotype: variants occurring upstream of amino acid 2154, disrupting both hepatic and intestinal ApoB isoforms (ApoB48/100), and variants affecting only ApoB100 were associated with cirrhosis, whereas the increased risk of HCC was exclusively observed for variants affecting ApoB48/100 [9]. Conversely, cardiovascular protection was more evident in carriers of ApoB100‐specific variants and appeared attenuated when both isoforms were disrupted [9].

Collectively, these findings support a model in which lifelong reduction of ApoB‐containing lipoproteins confers cardiovascular protection but, when driven by impaired lipoprotein secretion, predisposes to progressive liver disease [9]. These data advocate consideration of genetic screening for *APOB* variants in people with unexplained hypocholesterolemia and hepatic steatosis to predict the risk of liver disease progression, as well as in selected patients with cirrhosis or HCC to enable identification of at‐risk family members [9].

### Effect of loss of function variants in *MTTP* and liver disease

Evidence linking *MTTP* variants to liver disease remains limited and heterogeneous compared with *APOB* and derives mainly from rare disease descriptions and family‐based investigations and targeted association studies rather than large population‐scale sequencing analyses [52]. Biallelic *MTTP* LoF variants causing ABL represent the clearest biological model, in which defective VLDL assembly leads to extremely low circulating cholesterol and TG levels together with marked hepatic fat accumulation; however, current data are largely confined to case reports and small clinical series, precluding robust inference [8]. Evidence supporting a role of partial *MTTP* dysfunction outside classical ABL has emerged from family‐based studies [53]. In a four‐generation pedigree with progressive MASLD evolving to metabolic dysfunction‐associated steatohepatitis, cirrhosis, and HCC in the absence of metabolic risk factors, exome sequencing identified a causal *MTTP* variant (p.I564T) [53]. Functional experiments demonstrated reduced MTP protein levels, impaired ApoB secretion, intracellular TG accumulation, and activation of ER stress pathways, providing mechanistic evidence linking *MTTP* dysfunction to progressive liver disease [53].

Population‐based evidence remains sparse and mainly derives from candidate‐gene or cross‐sectional analyses. In a cross‐sectional study, including 1,193 participants, the *MTTP* Q297H polymorphism was associated with lower LDL‐C and non‐HDL‐C concentrations but an increased risk of MASLD after multivariable adjustment [54]. Similarly, a candidate‐gene sequencing study in 100 obese children reported that the *MTTP* rs2306986 variant was independently associated with MASLD susceptibility (OR 23; *p* = 0.044), although interpretation is limited by the small sample size and restriction to a high‐risk pediatric population [55]. Targeted resequencing in Japanese population cohorts further identified rare *MTTP* variants associated with MASLD susceptibility, with functional assays supporting impaired protein activity, but variant frequency was low and replication across ancestries remains limited [56].

Overall, available data indicate that impaired *MTTP*‐mediated lipoprotein secretion represents a biologically plausible mechanism promoting hepatic steatosis and progressive liver disease. However, current evidence relies predominantly on familial observations, selected cohorts, and cross‐sectional designs.

## Variants increasing LDL‐C catabolism: reduction in cardiovascular risk without risk for liver disease

A distinct group of monogenic hypocholesterolemia results from LoF variants that lower circulating lipoproteins by enhancing LDL‐C clearance rather than impairing hepatic VLDL assembly and secretion (Table 1) [8]. These conditions, primarily caused by pathogenic variants in *PCSK9* and *ANGPTL3*, reduce plasma lipoprotein concentrations through increased intravascular and receptor‐mediated catabolism, without the clear evidence of promoting intrahepatic lipid retention [57]. Biallelic LoF variants in *PCSK9* are exceedingly rare and lead to marked reductions in circulating LDL‐C, although precise lipid estimates in biallelic carriers remain limited due to their rarity, whereas TG levels and HDL‐C remain largely unchanged [58]. Heterozygous carriers represent the more common presentation and typically exhibit a 15%–28% reduction in LDL‐C, with mean concentrations consistently in the lower normal range, as originally demonstrated in population‐based and family studies of *PCSK9* deficiency [58]. LoF variants in *ANGPTL3* define familial combined hypolipidemia, a mechanistically related but phenotypically broader condition. Biallelic carriers display concomitant reductions in LDL‐C, HDL‐C, and TG frequently exceeding 50%–70%, with LDL‐C typically 1.46 ± 0.27 mmol/L, HDL‐C 0.67 ± 0.18 mmol/L, and TG approximately 0.54 mmol/L, reflecting enhanced lipoprotein catabolism mediated by disinhibition of both LPL and EL [59, 60]. Heterozygous carriers show a milder phenotype, with LDL‐C generally 2.75 ± 0.87 mmol/L and less pronounced reductions in HDL‐C and TG, as observed in large sequencing and population studies [61, 62].

### *PCSK9* loss of function variants reduce the risk of cardiovascular disease

Early evidence derives from family‐based and population genetic observations demonstrating that LoF variants in *PCSK9* reduce circulating LDL‐C without evidence of increased liver disease risk [58]. *PCSK9* LoF variants are relatively frequent compared with other monogenic hypocholesterolemic conditions, being present in approximately 1%–3% of adults, although prevalence varies by ancestry and variant type [63]. In a landmark population‐based analysis from the Atherosclerosis Risk in Communities, carriers of LoF variants in *PCSK9*, including nonsense mutations (Y142X and C679X), predominantly identified among African American participants, and the R46L missense variant, more frequently observed in European American participants, exhibited a 28% reduction in mean LDL‐C levels (*p* = 0.008) and an 88% lower risk of CHD among African American participants (HR 0.11; 95% CI 0.02–0.81; *p* = 0.03; age‐ and sex‐adjusted), whereas European American carriers showed a 15% reduction in LDL‐C associated with a 47% lower risk of coronary events (HR 0.50; 95% CI 0.32–0.79; *p* = 0.003), over a 15‐year follow‐up [58]. Concordantly with these data, in large population‐based cohorts, carriers of the *PCSK9* R46L variant exhibited a 15%–16% reduction in LDL‐C (0.35–0.55 mmol/L), corresponding to a 30% lower risk of ischemic heart disease (ORs ranging from 0.70 to 0.72 across different analytical models), an effect exceeding that predicted by the magnitude of LDL‐C reduction alone [64]. In population‐based exome sequencing analyses, LoF variants in *PCSK9* (and *ANGPTL3*) were associated with sustained reductions in LDL‐C without evidence of increased liver disease risk [61].

Consistently, genetic association studies in the UKBB and eMERGE cohorts indicated no association of *PCSK9* p.Arg46Leu variant with circulating liver enzymes and no association with MASLD (OR 0.89; 95% CI 0.55–1.42), despite chronically low LDL‐C concentrations [65]. Moreover, in an independent clinical cohort of people at risk of nonalcoholic steatohepatitis (NASH), carriers of *PCSK9* LoF variants were associated with a lower prevalence of hepatic steatosis, NASH, and advanced fibrosis (OR 0.42; 95% CI 0.22–0.81; *p* = 0.01; OR 0.48; 95% CI 0.26–0.87; *p* = 0.01; and OR 0.55; 95% CI 0.32–0.94; *p* = 0.03, respectively) [66]. Pharmacologic genetic concordance is observed in randomized evidence: A meta‐analysis of 25 trials, including 12,200 participants, showed evolocumab reduced abnormal liver tests compared with placebo (RR 0.43; 95% CI 0.20–0.93; *p* = 0.03) without hepatotoxicity [67], findings that are consistent with the overall hepatic safety profile reported in updated pooled analyses of *PCSK9* inhibitors [68]. Collectively, family observations, population genetics, and randomized trial data consistently indicate that lifelong reduction in LDL‐C levels driven by enhanced LDL clearance lowers CHD risk without increasing hepatic risk, with emerging evidence suggesting a potential reduction in hepatic steatosis and fibrosis in specific clinical settings [58, 64, 65, 66].

### *ANGPTL3* loss of function variants reduce cardiovascular disease

Population‐based resequencing studies demonstrated that rare variants in ANGPTL family members, including *ANGPTL3*, contribute to interindividual variability in plasma TG levels, with LoF variants enriched among individuals with lower TG levels, supporting a causal role of *ANGPTL3* in human TG metabolism [32]. Biallelic *ANGPTL3* LoF variants are very rare, with their precise prevalence remaining unknown; approximately 30 individuals from a limited number of families have been reported in the literature, although the condition is likely underdiagnosed [39]. Homozygosity for LoF variants in *ANGPTL3*, subsequently recognized as the genetic cause of familial combined hypolipidemia, is characterized by concomitant reductions in LDL‐C, HDL‐C, and TG resulting from enhanced intravascular lipoprotein catabolism rather than impaired hepatic secretion [59, 60]. Clinical characterization of affected families showed that homozygous or compound heterozygous carriers exhibit marked depletion of all major plasma lipoprotein classes, whereas heterozygous relatives display a milder but directionally concordant lipid phenotype [59, 60]. Population‐scale genetic analyses extended these observations and demonstrated that heterozygous *ANGPTL3* LoF variants are associated with lower LDL‐C and TG levels together with a reduced risk of CAD (OR 0.59; 95% CI 0.41–0.85; *p* = 0.004), confirming that lifelong lipid lowering mediated by enhanced intravascular lipolysis confers cardiovascular protection [31]. Despite experimental data suggesting increased intracellular lipid storage following *ANGPTL3* suppression, detailed clinical phenotyping has not shown evidence of liver disease [69]. People with complete or partial *ANGPTL3* deficiency exhibited comparable hepatic fat content to wild‐type controls (complete deficiency 9.8%, partial deficiency 10.1%, wild‐type 9.9%; *p* > 0.05) [69], and Mendelian randomization analyses confirmed no association between genetically determined *ANGPTL3* levels and hepatic fat accumulation [62]. Consistent with these findings, large exome sequencing datasets demonstrated persistent lipid reduction without enrichment for liver disease among carriers of *ANGPTL3* LoF variants [61]. Pharmacological studies further inform the hepatic effects of ANGPTL3 inhibition [70, 71, 72]. Monoclonal antibody‐mediated ANGPTL3 inhibition with evinacumab has demonstrated substantial LDL‐C reduction even in individuals with homozygous LDLR deficiency, indicating lipid lowering largely independent of LDLR activity, without evidence of hepatic lipid accumulation in clinical studies [70]. In the TRANSLATE‐TIMI 70 trial, treatment with the antisense oligonucleotide vupanorsen was associated with dose‐dependent increases in liver enzymes and hepatic fat fraction measured by magnetic resonance imaging‐proton density fat fraction (MRI‐PDFF) [71]. Elevations of alanine aminotransferase >3× the upper limit of normal occurred in up to 33.3% of participants and aspartate aminotransferase >3× the upper limit of normal in up to 44.4%, accompanied by a dose‐dependent increase in hepatic fat fraction of up to 76% relative to baseline [71]. In contrast, a phase 1 study of the hepatocyte‐targeted siRNA ARO‐ANG3 did not show worsening of hepatic steatosis. Among participants with baseline steatosis, liver fat measured by MRI‐PDFF showed a mean absolute change of −4.35% at Day 71 and −5.1% at Day 168, indicating no signal of hepatic fat accumulation [72]. Overall, available clinical evidence suggests that ANGPTL3 downregulation does not exert a consistent deleterious effect on hepatic fat accumulation [70, 71, 72].

## Differential diagnosis and diagnostic algorithm in case of hypocholesterolemia

Once secondary and acquired causes of hypocholesterolemia have been excluded, persistently low LDL‐C should be interpreted based on lipid concentration and assessment of the presence of liver steatosis. Although the magnitude of LDL‐C reduction and the accompanying lipoprotein profile, including the presence or absence of HDL‐C reduction [41], may provide initial diagnostic clues, clinical decision‐making should primarily rely on etiological classification, as the long‐term consequences of hypocholesterolemia are determined by the underlying biological mechanism rather than by absolute cholesterol levels. In this context, although no validated ApoB cut‐offs are currently established, markedly reduced ApoB levels in the presence of hepatic steatosis may support the interpretation of hypocholesterolemia [6]. Moreover, in clinical cohorts of people with MASLD, LDL‐C thresholds ≤55.4 mg/dL (1.4 mmol/L) have been shown to discriminate *APOB* variant carriers [9]. Once people with LDL‐C levels below this threshold and, where available, reduced ApoB levels have been identified, the presence of hepatic steatosis should be assessed by ultrasound, the first‐line imaging modality for steatosis detection, or by other available tools according to current guidelines, together with evaluation of liver injury through blood tests [73, 74].

In this setting, genetic testing may provide clinically relevant information by clarifying disease etiology, guiding longitudinal follow‐up, and enabling cascade evaluation of relatives (Fig. 2) [6].

**Fig. 2:**
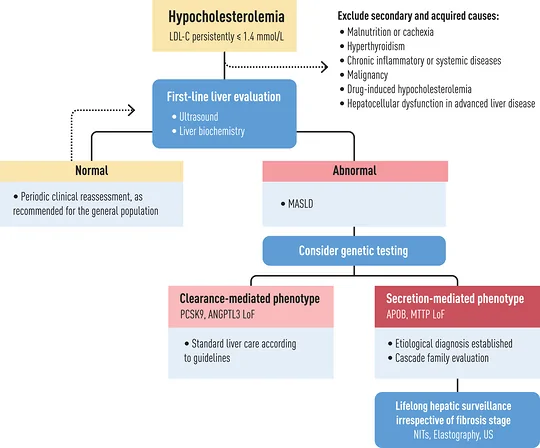
Diagnostic flowchart for the clinical evaluation of persistent hypocholesterolemia. In people presenting with persistently reduced LDL‐C levels (≤1.4 mmol/L) after confirmation on repeat testing and exclusion of secondary and acquired causes, first‐line liver evaluation includes US and liver biochemistry. People with normal imaging and laboratory findings should undergo periodic clinical reassessment, as recommended for the general population. In contrast, abnormal US findings and/or altered liver biochemistry should prompt evaluation for MASLD and consideration of genetic testing. Identification of secretion‐mediated disorders (APOB or MTTP LoF variants) establishes the etiological diagnosis, supports cascade family evaluation, and indicates liver disease staging and lifelong hepatic surveillance irrespective of fibrosis stage. Conversely, clearance‐mediated phenotypes (PCSK9 or ANGPTL3 LoF variants) are not associated with increased hepatic risk and should be managed according to current liver care guidelines. ANGPTL3, angiopoietin‐like protein 3; APOB, apolipoprotein B gene; LDL‐C, low‐density lipoprotein cholesterol; LoF, loss‐of‐function; MASLD, metabolic dysfunction‐associated steatotic liver disease; MTTP, microsomal triglyceride transfer protein; NITs, noninvasive tests; PCSK9, proprotein convertase subtilisin/kexin type 9; US, ultrasound.

In people without evidence of hepatic steatosis or liver injury, genetic testing is not routinely indicated, and follow‐up can be managed according to general population recommendations. Conversely, in people with hepatic steatosis in the context of low LDL‐C and ApoB, genetic testing should be considered to clarify the underlying mechanism. Subsequent management should be guided by the genetic result, distinguishing between hypocholesterolemia due to impaired secretion phenotype and clearance‐mediated phenotype.

In people with genetically confirmed pathogenic variants impairing hepatic ApoB‐containing lipoprotein secretion, genetic testing establishes the etiological diagnosis, guides longitudinal follow‐up, and enables cascade evaluation of relatives [6, 8, 9, 46]. Baseline risk stratification should be performed using validated noninvasive tests, including fibrosis scores, such as fibrosis‐4 index, or direct assessment of liver stiffness by elastography, to evaluate advanced fibrosis or cirrhosis, in line with contemporary noninvasive assessment strategies [73, 74]. These people should undergo lifelong surveillance irrespective of baseline fibrosis stage, given the variable penetrance and the potential for clinically silent progression [6, 8]. In the event of fibrosis progression, management should follow currently available therapeutic options, or those becoming available over time [75]. By contrast, current evidence does not support genotype‐driven lifelong surveillance in people with LoF variants in genes, such as *PCSK9* or *ANGPTL3*, in whom hypocholesterolemia results from enhanced lipoprotein clearance. In these individuals, liver‐related risk should be assessed and managed according to established noninvasive assessment strategies for steatotic liver disease, including baseline risk stratification and follow‐up based on fibrosis evaluation [73].

Taken together, this mechanism‐oriented diagnostic approach enables distinction between hypocholesterolemia reflecting impaired hepatic secretion phenotype and clearance‐mediated cardioprotective phenotypes, thereby supporting pathophysiology‐driven and individualized clinical management.

## Therapeutic implication

From a clinical perspective, lifestyle factors may contribute to the expression of liver disease in hypocholesterolemic states driven by impaired lipoprotein secretion. Diets rich in simple carbohydrates, particularly fructose and sucrose, may promote de novo lipogenesis and exacerbate hepatic steatosis [76], whereas alcohol exposure may act synergistically with impaired VLDL secretion, by further increasing intrahepatic TG accumulation and promoting inflammatory and fibrogenic injury, thereby potentially accelerating liver disease progression [77, 78]. These observations underscore the importance of careful lifestyle counseling in this setting, with particular emphasis on limiting simple carbohydrate intake and avoiding alcohol to mitigate disease progression [73].

From a therapeutic standpoint, targeting hepatic lipid handling may represent a rational strategy in this context (Fig. 3). In particular, therapeutic strategies should focus on limiting intrahepatic lipid accumulation rather than further reducing circulating lipoproteins. Therapeutic approaches may partially overlap with those used in MASLD, including agents targeting hepatic metabolism or promoting weight reduction, such as incretin‐based therapies. In this context, glucagon‐like peptide‐1 (GLP‐1) receptor agonists, such as semaglutide, and dual glucose‐dependent insulinotropic polypeptide/GLP‐1 receptor agonists, such as tirzepatide, may reduce hepatic steatosis largely through weight loss, improved insulin sensitivity, and favorable effects on metabolic dysfunction [79, 80, 81]. However, no dedicated studies have evaluated these therapeutic strategies in people with hypocholesterolemia due to impaired lipoprotein secretion. Approaches aimed at reducing de novo lipogenesis or improving hepatic lipid disposal, such as thyroid hormone receptor‐β agonists (THR‐β)—namely, resmetirom—offer a plausibly effective therapeutic hypothesis by enhancing hepatic fatty acid oxidation, increasing mitochondrial activity, and reducing intrahepatic TG content [75, 82]. These mechanisms may be particularly relevant in conditions characterized by impaired lipid export, where the therapeutic target is the intrahepatic lipid burden rather than circulating LDL‐C reduction. However, their efficacy and safety in this specific biological context remain to be established.

**Fig. 3:**
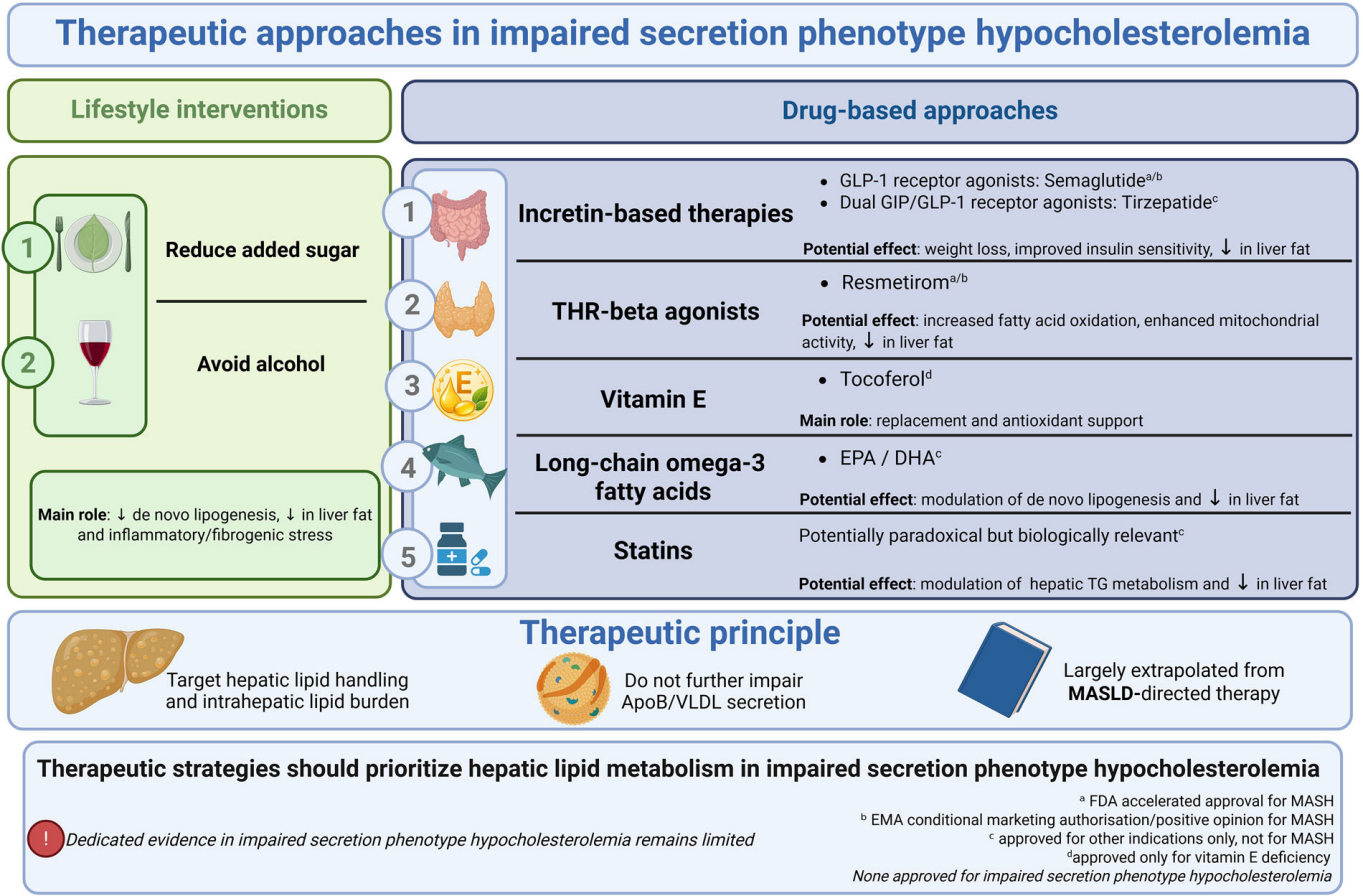
Clinical management and therapeutic perspectives in impaired secretion phenotype hypocholesterolemia. Therapeutic approaches in impaired secretion phenotype hypocholesterolemia should primarily aim to reduce intrahepatic lipid burden and improve hepatic lipid handling without further impairing ApoB/VLDL secretion. Lifestyle interventions, including reduction of added sugar intake and alcohol avoidance, may reduce de novo lipogenesis, intrahepatic TG accumulation, and inflammatory/fibrogenic stress. Drug‐based approaches may partially overlap with MASLD‐directed therapies and include incretin‐based therapies, THR‐β agonists, vitamin E supplementation, long‐chain omega‐3 fatty acids, and statins. These strategies remain largely extrapolated from MASLD or related metabolic settings, and dedicated evidence in impaired secretion phenotype hypocholesterolemia remains limited. Regulatory approval status is indicated for each agent with respect to MASH: ^a^ FDA accelerated approval for MASH; ^b^ EMA conditional marketing authorization or positive opinion for MASH; ᶜ approved for other indications only, not for MASH; ^d^ approved only for vitamin E deficiency. None of these agents is approved specifically for impaired secretion phenotype hypocholesterolemia. ApoB, apolipoprotein B; DHA, docosahexaenoic acid; EMA, European Medicines Agency; EPA, eicosapentaenoic acid; FDA, Food and Drug Administration; GIP, glucose‐dependent insulinotropic polypeptide; GLP‐1, glucagon‐like peptide‐1; MASH, metabolic dysfunction‐associated steatohepatitis; MASLD, metabolic dysfunction‐associated steatotic liver disease; TG, triglyceride; THR‐β, thyroid hormone receptor‐β; VLDL, very low‐density lipoprotein.

Vitamin E supplementation may also have a role, particularly in severe secretion‐driven hypocholesterolemias associated with impaired transport of fat‐soluble vitamins. In ABL and, to a lesser extent, FHBL, reduced circulating ApoB‐containing lipoproteins can limit vitamin E delivery to peripheral tissues, increasing susceptibility to oxidative injury and contributing to neurological and retinal complications [83, 84]. In this setting, vitamin E should be considered primarily as replacement therapy and antioxidant support rather than as a disease‐modifying treatment for hepatic steatosis. Whether vitamin E supplementation can modify liver disease progression in heterozygous secretion‐driven hypocholesterolemia remains unknown.

Long‐chain omega‐3 fatty acids may also represent a potential adjunctive approach, as they reduce hepatic TG content through modulation of de novo lipogenesis and key transcriptional regulators of lipid metabolism, such as sterol regulatory element‐binding protein 1c, carbohydrate‐responsive element‐binding protein, and peroxisome proliferator‐activated receptor pathways [85]. In a randomized controlled trial, treatment with docosahexaenoic acid and eicosapentaenoic acid (4 g/day) was associated with a numerical reduction in liver fat from 23% to 16.3%; however, this effect did not reach statistical significance in the intention‐to‐treat analysis (*β* = −3.64; *p* = 0.1), and no improvement in fibrosis scores was observed [86]. Notably, erythrocyte docosahexaenoic acid enrichment, reflecting biological exposure, was independently associated with a reduction in liver fat (*β* = −1.70 per 1% increase; *p* = 0.007), suggesting that adequate tissue exposure may be required to achieve a measurable hepatic effect [86]. Thus, omega‐3 fatty acids may be considered biologically plausible but have not been specifically evaluated in impaired secretion phenotype hypocholesterolemia.

A potentially paradoxical but relevant therapeutic approach could involve statins, as inhibition of hepatic cholesterol synthesis has been shown to reduce VLDL production, a process tightly linked to hepatic TG availability [87, 88]. In a randomized placebo‐controlled study, high‐dose atorvastatin (80 mg/day) was associated with an approximately 40% reduction in liver TG mass in pooled liver samples, together with significant downregulation of sterol regulatory element‐binding protein 1c and ANGPTL3, key regulators of hepatic TG metabolism [89]. In line with these mechanistic observations, in matched observational analyses of at‐risk individuals, statin use was associated with protection across the full spectrum of liver damage, including steatosis (aOR 0.09 [0.01–0.32]), steatohepatitis (0.25 [0.13–0.47]), and fibrosis stage F2–F4 (0.42 [0.20–0.85]; all *p* < 0.05), with evidence of a dose‐dependent effect [90]. Notably, the protective association with steatohepatitis was attenuated in carriers of the *PNPLA3* I148M variant, suggesting that genetic background may modulate treatment response [90]. Taken together, these data suggest that statins may modulate hepatic steatosis and liver injury through mechanisms extending beyond LDL‐C reduction. However, whether this effect translates to hypocholesterolemic states driven by impaired lipoprotein secretion, and whether it differs according to genetic background, requires dedicated evaluation in targeted studies.

By contrast, strategies that directly reduce ApoB synthesis or MTP activity should be interpreted with caution in this setting. Although ApoB‐targeting antisense therapy and MTP inhibition have been developed to lower LDL‐C in severe hypercholesterolemia, both approaches reduce hepatic lipoprotein export and have been associated with hepatic TG accumulation or steatosis in clinical studies [91, 92, 93]. Therefore, in impaired secretion phenotype hypocholesterolemia, these pathways currently represent mechanisms to avoid exacerbation rather than therapeutic targets, unless future studies identify context‐specific strategies able to improve hepatic lipid handling without further impairing ApoB‐containing lipoprotein secretion.

## Conclusion

Hypocholesterolemia should not be interpreted solely as a benign metabolic finding but rather as a potential etiological signal whose clinical implications depend on the underlying biological mechanism. In people in whom hypocholesterolemia coexists with liver steatosis, targeted genetic testing may help identify forms related to impaired hepatic secretion of ApoB‐containing lipoproteins. Conversely, hypocholesterolemia driven by enhanced lipoprotein clearance represents a biologically distinct phenotype without adverse hepatic consequences. Distinguishing between these mechanisms allows focused evaluation of people with hypocholesterolemia and liver involvement while avoiding unnecessary investigations in clearance‐mediated cardioprotective phenotypes, supporting a pathophysiology‐driven and clinically relevant interpretation of low LDL‐C.

## Author contributions

Stefano Romeo conceived the manuscript. Valentina Flagiello wrote the first manuscript draft. Stefano Romeo and Umberto Vespasiani‐Gentilucci critically revised the manuscript for important intellectual content and supervised the work. Emelie Barreby contributed to manuscript revision. All authors approved the final version of the manuscript.

## Conflict of interest statement

S.R. reports research support for the present work from the Swedish Cancerfonden, the Swedish Research Council (Vetenskapsrådet), the Swedish state under the ALF agreement, the Swedish Heart Lung Foundation, the Novo Nordisk Distinguished Investigator Grant—Endocrinology and Metabolism, Novo Nordisk Project Grants in Endocrinology and Metabolism, and a Novo Nordisk donation to the Karolinska Institutet in connection with his professor appointment. S.R. received a research grant for studies on molecular genetics of MASLD. S.R. reports consulting fees from AstraZeneca, GSK, Celgene Corporation, Ribocure AB, Madrigal, Ultragenyx, Sanofi, Wave Life Sciences, Lipigon, Novartis, Profluent, Airna, Echosense, and Chiesi; equity in Heptabio; and inventorship on a patent entitled “Method for treating fatty liver disease” (US application number 17/480266). U.V.‐G. reports consulting fees from Ipsen, Gilead, Madrigal, Novo Nordisk, Mirum, GSK, Advanz, and AstraZeneca, with payments made to him and, for Gilead and AstraZeneca, also to his institution. V.F. and E.B. declare no conflicts of interest.
